# Exposure to phthalates and bisphenol A are associated with atopic dermatitis symptoms in children: a time-series analysis

**DOI:** 10.1186/s12940-017-0225-5

**Published:** 2017-03-09

**Authors:** Eun-Hye Kim, Byoung-Hak Jeon, Jihyun Kim, Young-Min Kim, Youngshin Han, Kangmo Ahn, Hae-Kwan Cheong

**Affiliations:** 1 Department of Social and Preventive Medicine, Sungkyunkwan University School of Medicine, 2066 Seobu-ro, Jangan-gu, Suwon, Gyeonggi-do 16419 Republic of Korea; 2 Samsung Biomedical Research Institute, Sungkyunkwan University School of Medicine, Seoul, 06351 Republic of Korea; 3 Department of Pediatrics, Samsung Medical Center, Sungkyunkwan University School of Medicine, Seoul, 06351 Republic of Korea; 4 Environmental Health Center for Atopic Diseases, Samsung Medical Center, Seoul, 06351 Republic of Korea

**Keywords:** Endocrine disrupting compounds, Atopic dermatitis, Phthalate, Bisphenol A, Panel study

## Abstract

**Background:**

Despite increasing evidence on the relationship between exposure to phthalates and bisphenol A with allergies and asthma, reports on atopic dermatitis (AD) with these chemicals are few. We assessed the association between AD symptoms and the exposure to phthalates and bisphenol A and in children.

**Methods:**

We surveyed 18 boys with AD (age 3–7 years) in a day care center in Seoul between May 2009 and April 2010. AD symptoms were recorded by using a daily symptom diary. We collected 460 series of pooled urine twice a day, in the morning and afternoon, over 230 working days and measured the concentrations of mono–2–ethyl-5-oxohexyl phthalate (5-oxo-MEHP), mono-2-ethyl-5-hydroxyhexyl phthalate (5-OH-MEHP), mono-isobutyl phthalate (MnBP) and bisphenol A glucuronide (BPAG) in the pooled urine. Logistic regression was used for statistical analysis.

**Results:**

Most phthalate metabolite levels were higher in the morning than in the afternoon (*p* < 0.0001). There was seasonal variation in the levels of phthalates and bisphenol A metabolites. Levels of 5-OH-MEHP, MnBP, and BPAG were highest in summer (*p* < 0.0001). Manifestation of AD symptoms was associated with an increase in urinary levels of MnBP (adjusted odds ratio, aOR = 2.85, 95% CI: 1.12-7.26 per 1 μg/L of MnBP) and BPAG (aOR = 1.79, 95% CI: 0.91-3.52 per 1 μg/L BPAG) on the same day. The levels of MnBP and BPAG in the previous day increased AD symptoms (aOR = 2.74, 95% CI: 1.21-6.20, for 1 μg/L of MnBP and aOR = 2.01, 95% CI: 1.08-3.74 for 1 μg/L BPAG).

**Conclusion:**

Our results suggest that exposure to phthalates and bisphenol A is associated with aggravation of AD symptoms in children.

## Highlights

1. Pooled urine can be a useful tool for the long-term monitoring of chemical exposures in a confined environment.
2. Phthalates and bisphenol A exposure is related with the aggravation of atopic dermatitis symptom in children.

## Background

Phthalic acid esters (phthalates) and bisphenol A (BPA) are widely used endocrine disrupting chemicals (EDCs) [26, 29]. Phthalates are found in a wide range of indoor environment, implying that people can be exposed to phthalates through inhalation and dermal absorption [7]. BPA is commonly used in food or beverage cans, electronic products, and household products such as water bottles, baby bottles, dental fillings, and dental sealants [36].

Previous studies reported that PVC flooring in the parents’ bedrooms was associated with the development of asthma among preschool children. In addition, effects of early life phthalate exposure on immunoglobulin E (IgE) levels can potentially cause atopic dermatitis (AD) in children in a 10-year cohort [32, 37]. Indoor dust that includes di-2-ethylhexyl phthalate (DEHP) is associated with wheezing in preschool-aged children [6, 23]. Moreover, another study has reported that higher levels of BBzP and DiBP in house dust increase OR of atopic dermatitis among children [2]. During the prenatal period and early childhood, exposure to BBzP may influence the risk of developing eczema [19]. The associations between urinary BPA concentrations and asthma were also addressed in children from a birth cohort study [10].

Children are especially vulnerable to indoor environmental hazards because they breathe more air per kilogram of body weight than adults [35] and spend a longer time at indoor facilities such as school, kindergarten, or day care centers. Recently, human biological monitoring data showed that the tolerable intake for children has been exceeded to a considerable degree [41]. Children showed clearer association of atopic dermatitis with BBzP and DiBP in house dust than adults [2] and daily phthalate intakes estimated from urinary phthalate metabolites are higher in children than adults [3].

AD is the most prevalent type of allergic disorder in early childhood. In Korea, the prevalence of AD in children less than 24 months of age has increased from 19.8% in 2003 to 23.8% in 2008 [46]. AD has become one of the most prevalent health problems of children from an environmental health perspective. Although increasing evidence supports that exposure to phthalates and BPA is linked with allergies and asthma [11, 27, 29, 39], there are still insufficient data about the effect of exposure to phthalates and BPA on AD symptoms in a longitudinal study. In this study, we investigated the associations between daily levels of phthalates and BPA metabolites in urine and AD symptoms using a time series analysis.

## Methods

### Study design and participants

This study was based on a panel study involving a time series of repeated measurements of health outcome and exposure. Between May 2009 and April 2010, we selected a day care center authorized to take care of children with AD located in the northeast region of Seoul. It is located near the metro station, but traffic is relatively sparse. There are no identified sources of pollution such as factories or incinerators nearby.

Eighteen boys with AD aged 3 to 7 years were recruited for this study. Their mean (standard deviation, SD) age was 4.3 ± 0.8 years. The diagnosis of AD was determined according to the Hanifin and Rajka’s diagnostic criteria, which requires presence of at least three of four major features and at least three of 23 minor features in the physical signs of AD [12] after all children were examined by a pediatric allergist who regularly visited the day care center to follow up on the children's health status. This study was approved by the Institutional Review Board of Samsung Medical Center (SMC 2013-04-057). Informed consent was obtained from the parents of all participating children.

### Atopic dermatitis symptoms

A diary was developed to measure the severity of AD symptoms [20]. The diary included levels of pruritus during daytime, daily activity schedules, and changes in indoor environment status (presence of an air conditioner, ventilator, air cleaner, or humidifier). Daily pruritus level was assessed using a VAS scale ranging between 0 and 10. The symptom diary was recorded by teachers who were trained on the evaluating and recording symptoms prior to the study. They were instructed to record once a day for each child with AD. At the end of each week, the diaries were returned to the research assistant and were thoroughly scrutinized for any missing or error records.

As we used pooled urine as a whole to measure the levels of each metabolite for each day, we applied an AD symptom manifestation rate (ASMR) to evaluate severity of daily level of AD symptom in group as a matching measurement of health outcome. The AD symptom is first classified into two categories: 0 = No AD symptom or 1 = presence of AD symptoms (pruritus level greater than 2) for each boy. We then summed the cases with AD symptom and divided by total number of attendants to the day care center for each day. The formula for ASMR is as follows:

$$ \mathrm{AD}\kern0.5em \mathrm{symptom}\kern0.5em \mathrm{manifestation}\kern0.5em \mathrm{rate}=\frac{\mathrm{Sum}\ \mathrm{of}\ \mathrm{daily}\ \mathrm{AD}\ \mathrm{cases}\ \mathrm{with}\ \mathrm{manifested}\ \mathrm{symptom}}{\mathrm{Sum}\ \mathrm{of}\ \mathrm{daily}\ \mathrm{AD}\ \mathrm{cases}\ \mathrm{attended}\ \mathrm{in}\ \mathrm{the}\ \mathrm{child}\ \mathrm{care}\ \mathrm{center}} $$

### Urine collection and chemical analyses

The urine from 18 boys was collected and pooled into a collecting bottle installed in a boys’ toilet on the same floor of the daily classroom twice a day during the study period. We used boys’ urine for the sustainability and convenience of collection and pooled the collected urine considering cost-effectiveness, which has been adopted in previous studies [14–16, 38, 45, 47]. On a daily basis, individual urine from 18 boys was continuously combined to make two pools: morning (9:00–11:00 am) and afternoon (2:00–4:00 pm). The pooled urine was collected by teachers every morning and afternoon in non-vinyl and non-polycarbonate containers, frozen immediately, sent to the laboratory for testing within 1 to 5 h, and stored at below −70 °C for a maximum of six months before analysis.

The urine samples were analyzed for three phthalates and one BPA metabolite. We chose mono-n-butyl phthalate (MnBP) from di(n-butyl) phthalate (DnBP) and BBzP as primary metabolites and mono-2-ethyl-5-oxohexyl phthalate (MEOHP), mono-2-ethyl-5-hydroxyhexyl phthalate (MEHHP) from di(2-ethylhexyl) phthalate (DEHP), and bisphenol A glucuronide (BPAG) as secondary metabolites. The targeted phthalates and BPA metabolites were analyzed by high performance liquid chromatography (HPLC, Agilent 1200 series, Agilent Technologies, Santa Clara, CA, USA) with a tandem mass spectrometer (Agilent Triple Quad 6410, Agilent Technologies, Santa Clara, CA, USA) following the method published by the Centers for Disease Control and Prevention Laboratory Procedure Manual [9] including the quality control system, handling, and analysis of samples. Internal quality control was performed by analyzing control urine with known concentrations. External quality assurance was provided by the German External Quality Assessment Scheme for Biological Monitoring (G-EQUAS) in the case of metabolites of phthalates. The limits of detection were as follows: 0.6 μg/L for MnBP, 0.5 μg/L for MEOHP, 0.4 μg/L for MEHHP and 0.15 μg/L for BPA.

### Statistical analyses

Urine data from children were compared in terms of metabolite levels of phthalates and BPA between the urine collected in the morning and afternoon by season using the geometric mean with geometric standard error. Because of the skewed distributions, phthalate and BPA metabolite levels were log-transformed before analyses. All log-transformed data were normally distributed and no significant outliers were found.

We considered days with over 50% of ASMR as having severe AD symptom incidence and days with less than 50% of ASMR as having mild or negligible incidence. Differences in phthalates and BPA metabolite levels between the morning and afternoon were examined by the Student’s t-test. Comparisons of metabolite levels by season were done by chi-square analysis.

The associations between ASMR and phthalates and BPA metabolites were analyzed by multiple logistic regression models. The goodness of fit of a model was assessed using a likelihood ratio chi-square. We adjusted for daily temperature, daily relative humidity, and seasonal effects. The specification of the models was as follows:

$$ \mathrm{Logit}\left[\mathrm{P}\left(\mathrm{y}=1\right)\right]={\beta}_0+{\beta}_1(metabolite)+{\beta}_2(temperature)+{\beta}_3\left( relative\  humidity\right)+{\upbeta}_4(season) $$

where y is the daily ASMR and *β*_1_ is daily level of each targeted phthalate and a BPA metabolite. Lag effects of metabolites of phthalates and BPA on ASMR were estimated up to three days. Unlagged (Lag0) designates the effect of each targeted metabolite on the current day on ASMR and Lag1 ~ Lag3 designates the effect of metabolite on 1 to 3 days in advance on ASMR. The significance level was set at 0.05 as the standard. SAS version 9.3 (SAS Institute, Inc., Cary, NC, USA) was used for all statistical analyses.

## Results

The characteristics of the children with AD symptoms determined in each season were summarized in Table 1. ASMR at each season (spring, summer, autumn, winter) were 29.1, 57.2, 53.5, and 56.9%, respectively, highest in summer and lowest in spring. The difference in the ASMR was statistically significant between seasons (*p* < 0.0001).

**Table 1:** Summary of atopic dermatitis symptoms in each season

	Spring	Summer	Fall	Winter	Year-round
Days observed	57	53	64	56	230
Daily attendance in child care center (Mean ± SD)	7.2 ± 2.1	5.2 ± 2.0	8.0 ± 2.2	6.0 ± 2.3	6.7 ± 2.4
No. of children per day with atopic dermatitis (Mean ± SD)	2.1 ± 0.9	3.0 ± 1.9	4.6 ± 2.3	3.5 ± 1.6	3.4 ± 2.0
Mean daily ASMR (%)^*^	29.1	57.2	53.5	56.9	49.5
	b^a^	a	a	a	

^*^ *p* < .0001 by ANOVA between atopic dermatitis symptoms and season

^a^Duncan’s post hoc multiple comparison for each season in the ANOVA. The difference between Group A and Group B is significant

ASMR: atopic dermatitis symptom manifestation rate

**Fig. 1:**
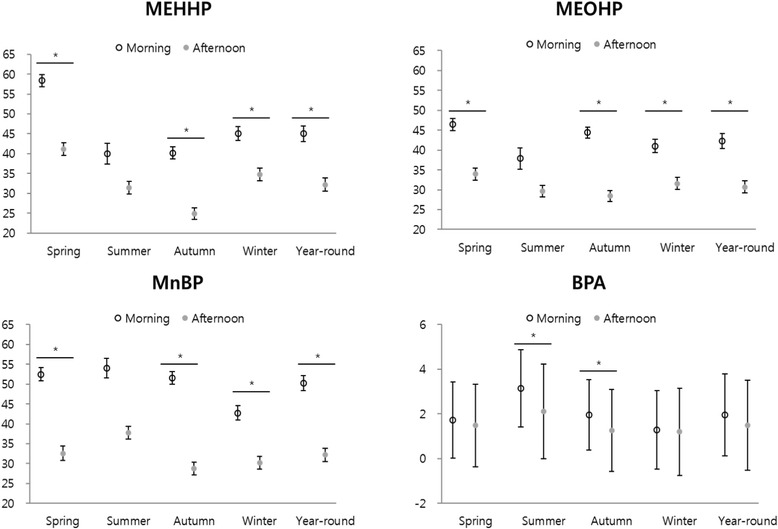
Geometric mean and standard deviation of metabolites of phthalates and bisphenol A in the morning and afternoon urine by season. * Significant by the Student’s t-test at *p-*value = 0.05

During the 12 months of the study period, 460 urinary concentration data points were available, consisting of 230 data points for each morning and afternoon. Figure 1 shows differences in phthalate and BPA metabolite levels in urine between the morning and afternoon by season. Phthalate metabolites (MEHHP, MEOHP, and MnBP) were significantly higher in the morning than in the afternoon (*p* < 0.0001) except in summer for MEHHP and MEOHP. Morning levels of BPAG metabolite were higher than those of the afternoon in summer and autumn (*p* < 0.0001), but their differences were less prominent compared to those of the phthalates metabolites. The geometric mean (GM) (geometric standard deviation (GSD)) of MEOHP, MEHHP and MnBP concentrations for daily average were 71.97 (1.64), 68.11 (1.58) and 76.02 (1.71) μg/g creatinine at daily average. The GM (GSD) in the morning were 77.03 (1.83), 72.34 (1.78), 85.97 (1.82) and 3.36 (1.9), μg/g creatinine in the afternoon were 67.24 (1.41), 64.13 (1.33), 67.23 (1.54) and 3.09 (2.07) μg/g creatinine for MEOHP, MEHHP, MnBP and BPA, respectively.

Pearson correlation coefficients among ASMR and phthalates and BPA metabolites are shown in Table 2. Secondary metabolites (MEHHP, MEOHP) were significantly correlated with the primary metabolite (MnBP) (R = 0.71 and 0.77). The secondary metabolites of DEHP (MEHHP and MEOHP) were strongly correlated with each other (R = 0.92). All urinary levels of phthalate metabolites, DEHP and DnBP, were positively correlated with BPAG levels (R = 0.20, 0.26, and 0.42 for MEHHP, MEOHP, and MnBP, respectively). There was significant correlation between the presence of ASMR and MnBP (R = 0.16, *p* = 0.0231) and BPAG levels (R = 0.16, *p* = 0.0243).

**Table 2:** Correlation between atopic dermatitis symptom manifestation rate (ASMR) and metabolites of phthalates and bisphenol A

	ASMR	MEHHP	MEOHP	MnBP	BPA
ASMR	1				
MEHHP	−0.0909	1			
MEOHP	0.0445	0.9165^c^	1		
MnBP	0.1578^a^	0.7111^c^	0.7785^c^	1	
BPAG	0.1566^a^	0.2039^b^	0.2634^c^	0.4243^c^	1

ASMR: Atopic dermatitis symptom manifestation rate

MEHHP: Mono-2-ethyl-5-hydroxyhexyl phthalate

MEOHP: Mono-2-ethyl-5-oxohexyl phthalate

MnBP: Mono-n-butyl phthalate

BPAG: Bisphenol A glucuronide

^a^ *p*-value < 0.05, ^b^ *p*-value < 0.01, ^c^ *p*-value < 0.001 (extremely significant)

Table 3 presents the influence of phthalates and BPA metabolites on ASMR after controlling for temperature, humidity, and season. ASMR was significantly associated with MnBP and BPAG concentrations. Manifestation of AD symptoms was strongly associated with an increase of urinary MnBP levels on the same day (adjusted odds ratio (aOR) = 2.85, 95% CI: 1.12-7.26). There were no significant relationships between MEOHP, MEHHP, and BPAG and AD symptoms. In lagged models, the previous day’s MnBP and BPAG in urine were positively associated with ASMR (aOR = 2.74, 2.01, 95% CI: 1.21-6.20, 1.08-3.74, respectively) and MEOHP levels in urine two days before were positively associated with ASMR (aOR = 3.11, 95% CI: 1.01-9.61).

**Table 3:** Adjusted odds ratios (95% CI) between atopic dermatitis symptoms and urine concentration of phthalates and bisphenol A metabolites

Metabolite	Lag^b^ 0			Lag 1			Lag 2			Lag 3		
	aOR^a^	95%CI		aOR	95%CI		aOR	95%CI		aOR	95%CI	
MEHHP	1.21	0.47	3.07	1.46	0.57	3.70	1.82	0.71	4.66	2.13	0.82	5.53
MEOHP	2.28	0.74	7.00	1.96	0.75	5.10	3.11	1.01	9.61	2.98	0.96	9.27
MnBP	2.85	1.12	7.26	2.74	1.21	6.20	2.02	0.82	4.97	1.71	0.69	4.21
BPAG	1.79	0.91	3.52	2.01	1.08	3.74	1.63	0.84	3.19	1.53	0.78	2.98

^a^aOR: Adjusted for temperature, humidity, and season

^b^Lag *x*: Effect of urine concentration of phthalates and bisphenol A metabolites *x* day(﻿s) ahe﻿ad﻿ on atopic dermatitis symptoms

## Discussion

We investigated the effects of phthalates and BPA on manifestation of AD symptoms in children attending a day care center based on daily measurements over a year. AD symptoms in children were associated with phthalates and BPA metabolites. AD symptoms were strongly associated with MnBP and BPAG in both unlagged and one day-lagged models with statistical significance (Table 3).

Previous studies have reported that associations between MEHP levels, higher-molecular-weight phthalates such as DEHP, and serum IgE levels in boys are related to an increase in IgE sensitization [17, 37]. In addition, MnBP was significantly associated with AD. A subcutaneous injection of MEHP increased IgE levels in mice, and subcutaneous injection of phthalate can aggravate AD-like skin lesions induced by *Dermatophagoides pteronyssinus* in mice [25, 34]. Animal and *in vitro* studies suggested a possible role of BPA in the pathogenesis of allergic disease; urinary BPA is significantly associated with allergic asthma, as it might act as an allergic or TH2 sensitizer and induce specific IgE responses (National Health and Nutrition Examination Survey 2005–2006) [36, 44].

The major route of exposure to DEHP and BPA is dietary intake, and urine levels tend to be lower during the fresh foods intervention, which implies that phthalate intake can be reduced by avoiding foods packaged, stored, or heated in plastic containers [28, 31]. Although dietary ingestion is the primary route of human exposure to BPA, it was detected in most children’s solid and liquid food, followed by indoor air, hard floor surface wipe, food preparation surface wipe, and transferable residue samples [40]. Routes of exposure to phthalates have multiple pathways and sources. Phthalates were mostly detected in a wide range of food, particularly high fat food. Ingestion through house dust and hand–to-mouth transfer of products was reported in addition to dermal intake from cosmetics and personal care products [7, 27, 35, 42].

Although most information on exposure to phthalates has focused on food and water, inhalation intake should not be neglected. Children spend a large fraction of their time indoors, and air concentrations of phthalates are approximately 10 times higher in indoor air than in outdoor air [29]. Not only BBzP, DEHP, and DnBP, but also DiNP in house dust increase in the dwellings with PVC wall and floor [1]. Furthermore, the levels of phthalate metabolites such as BBzP and DEHP in house dust were associated with PVC flooring, and high concentrations of DEHP were associated with buildings constructed before 1960 [8]. Bekö et al. [7] reported that indoor environmental exposures for diethyl phthalate (DEP), DnBP and diisobutyl phthalate (DiBP) is meaningful fraction of total daily intake through inhalation and dermal absorption in children. For DEP, DnBP and DiBP, exposures to air and dust in the indoor environment accounted for approximately 100, 15 and 50% of the total intake, respectively.

Ait Bamai et al. [3] found the daily phthalate intakes estimated from urinary metabolites were higher in children than their parents. For infants and toddlers, one of the most important phthalate intake sources is floor dust. Young children ingest up to 10 times more house dust than adults, and are therefore more vulnerable to exposure to phthalates because of their hand-to-mouth behavior on the flooring [1, 21]. House dust is also well known to be a main exposure source to allergens and other EDCs for infants. Similar to indoor air concentrations, levels of phthalates in settled dust have been associated with PVC building materials [3, 8]. Yamamoto [43] found substantial migration of BPA from PVC hoses into room temperature water. BPA exposure from this source by ingestion or inhalation deserves consideration because PVC pipe is approved for use in residential water supply lines. Hsu et al. [18] examined the home environment along with bio-monitoring data. They noted that exposure to indoor dust-borne BBzP, DBP, and the metabolites MBP and MEHP in urine from children aged 3 to 9 years were associated with increased risk of having allergy, asthma and related symptoms. Prenatal urinary BPA was associated with increased odds of wheezing in early life [33].

The Geometric SDs of urinary levels of phthalates and BPA metabolites in the morning were higher than those in the afternoon. These results may be associated with the time at which the children performed certain activities. The urinary metabolite levels in the morning may reflect more exposure to phthalates at home than that in the day care center, while those in the afternoon may reflect exposure at day care centers when considering the half-life of phthalates in body. Most orally-administered phthalates and BPA are systemically absorbed and excreted in urine. The elimination half-lives for secondary metabolites of DEHP such as MEOHP and MEHHP have been estimated as 10–24 hours in healthy male volunteers; after 24 hours, 67.0% of the DEHP dose was excreted in urine [22, 30].

The p-values for the correlations listed in Table 2 were significant for all metabolites. As expected [4, 13, 24], the secondary metabolites (MEHHP and MEOHP) were strongly correlated each other. Note that each metabolite of phthalates has a different phthalate exposure source. The strong correlations between phthalate metabolites imply that the children in our study are exposed to a mixture of phthalates every day. The correlations among the primary metabolite of DnBP (MnBP) and secondary metabolites of DEHP (MEHHP and MEOHP) were also strong, which also indicates the use of mixtures of phthalates in products [5].

This study has limitations in that exposure and health outcome are not individually matched. Urinary levels of metabolites from pooled urine have been used for studies of environmental exposure assessment in previous studies [14–16, 38, 45, 47]. Our measurement of exposures to phthalates and BPA metabolites for over a year in a limited population of children sharing the same space and life style during the day may provide a rationale for a time series analysis of risk factors for the manifestation of AD symptom aggravation. Geometric SDs of the metabolite level were narrow, reflecting a relatively small range of variability between individuals. Despite the relatively small number of subjects, repeated measures of exposure and health outcome over one year period by a simple but consistent method yielded statistical significance.

To our knowledge, this is the first longitudinal study to report an association between urinary phthalates and BPA concentrations and AD symptoms in children based on daily measurements. This study includes long-term AD symptom records and urine biomarker levels covering four consecutive seasons, which enabled us to assess the exposure to phthalates and BPA by season and time lag effects. The results of this study provide evidence that exposure to EDCs such as phthalates and BPA can be a risk factor for AD symptom aggravation, demonstrating that to reduce AD symptoms, children with AD should be prevented from exposure to phthalates and BPA from plasticized products.

## Conclusion

Our study results suggest that exposure to phthalates and BPA is a risk factor for AD symptom aggravation in children. As plasticized products have become ubiquitous in homes and day care centers, more efforts are required to reduce exposure to phthalates and BPA to avoid aggravation of AD symptoms, especially in children. Further studies on sources and assessment of EDC exposure in children, especially regarding socioeconomic status and time-activities are required to better understand the effect of EDCs on AD and to devise strategies to reduce their intake.

## Abbreviations

5-OH-MEHP: Mono-2-ethyl-5-hydroxyhexyl phthalate
5-oxo-MEHP: Mono-2-ethyl-5-oxohexyl phthalate
AD: Atopic dermatitis
aOR: Adjusted odds ratio
ASMR: Atopic dermatitis symptom manifestation rate
BBzP: Benzyl butyl phthalate
BPA: Bisphenol A
BPAG: Bisphenol A glucuronide
CI: Confidence interval
DBP: Dibutyl phthalate
DEHP: Di-2-ethylhexyl phthalate
DiBP: Diisobutyl phthalate
DnBP: Di(n-butyl) phthalate
EDCs: Endocrine disrupting compounds
IgE: Immunoglobulin E
MEHHP: Mono-2-ethyl-5-hydroxyhexyl phthalate
MnBP: Mono-n-butyl phthalate
SD: Standard deviation
VAS: Visual analogue scale

## References

[1] Ait Bamai Y, Araki A, Kawai T, Tsuboi T, Saito I, Yoshioka E, Kanazawa A, Tajima S, Shi C, Tamakoshi A, Kishi R (2013). Associations of phthalate concentrations in floor dust and multi-surface dust with the interior materials in Japanese dwellings. Sci Total Environ.

[2] Ait Bamai Y, Shibata E, Saito I, Araki A, Kanazawa A, Morimoto K, Nakayama K, Tanaka M, Takigawa T, Yoshimura T, Chikara H, Saijo Y, Kishi R (2014). Exposure to house dust phthalates in relation to asthma and allergies in both children and adults. Sci Total Environ.

[3] Ait Bamai Y, Araki A, Kawai T, Tsuboi T, Saito I, Yoshioka E, Cong S, Kishi R (2016). Exposure to phthalates in house dust and associated allergies in children aged 6-12years. Environ Int.

[4] Baird DD, Saldana TM, Nepomnaschy PA, Hoppin JA, Longnecker MP, Wein-berg CR, Wilcox AJ (2010). Within-person variability in urinary phthalate metabolite concentrations: measurements from specimens after long-term frozen storage. J Expo Sci Environ Epidemiol.

[5] Becker K, Göen T, Seiwert M, Conrad A, Pick-Fuß H, Müller J, Wittassek M, Schulz C, Kolossa-Gehring M (2009). GerES IV: phthalate metabolites and bisphenol A in urine of German children. Int J Hyg Environ Health.

[6] Bekö G, Callesen M, Weschler CJ, Toftum J, Langer S, Sigsgaard T, Høst A, Kold Jensen T, Clausen G (2015). Phthalate exposure through different pathways and allergic sensitization in preschool children with asthma, allergic rhinoconjunctivitis and atopic dermatitis. Environ Res.

[7] Bekö G, Weschler CJ, Langer S, Callesen M, Toftum J, Clausen G (2013). Children’s phthalate intakes and resultant cumulative exposures estimated from urine compared with estimates from dust ingestion, inhalation and dermal absorption in their homes and daycare centers. PLoS One.

[8] Bornehag CG, Lundgren B, Weschler CJ, Sigsgaard T, Hagerhed-Engman L, Sundell J (2005). Phthalates in indoor dust and their association with building characteristics. Environ Health Perspect.

[9] CDC (Centers for Disease Control and Prevention) (2009). Phthalate Metabolites in Urine, NHANES 2005–2006. Laboratory Procedure Manual Centers for Disease Control and Prevention. Atlanta, GA: Centers for Disease Control and Prevention.

[10] Donohue KM, Miller RL, Perzanowski MS, Just AC, Hoepner LA, Arunajadai S, Canfield S, Resnick D, Calafat AM, Perera FP, Whyatt RM (2013). Prenatal and postnatal bisphenol A exposure and asthma development among inner-city children. J Allergy Clin Immunol.

[11] Gascon M, Casas M, Morales E, Valvi D, Ballesteros-Gómez A, Luque N, Rubio S, Monfort N, Ventura R, Martínez D, Sunyer J, Vrijheid M (2014). Prenatal exposure to bisphenol A and phthalates and childhood respiratory tract infections and allergy. J Allergy Clin Immunol.

[12] Hanifin JM, Rajka G (1980). Diagnostic features of atopic dermatitis. Acta Derm Venereol.

[13] Hatch EE, Neslon JW, Qureshi MM, Wienberg J, Moore LL, Singer M, Webster TF (2008). Association of urinary phthalate metabolite concentration with body mass index and waist circumference: a cross-sectional study of NHANES data,1999–2002. Environ Health.

[14] Heffernan A, Aylward L, Toms LML, Eaglesham G, Hobson P, Sly P, Mueller JF (2013). Age-related trends in urinary excretion of bisphenol A in Australian children: evidence from a pooled sample study using samples of convenience. J Toxic Environ Health A.

[15] Heffernan AL, Sly PD, Toms LM, Hobson P, Mueller JF (2014). Bisphenol A exposure is not associated with area-level socioeconomic index in Australian children using pooled urine samples. Environ Sci Pollut Res Int.

[16] Heffernan A, Aylward L, Toms LML, Sly P, MacLeod M, Mueller JF (2014). Pooled biological specimens for human biomonitoring of environmental chemicals: opportunities and limitations. J Expo Sci Environ Epidemiol.

[17] Heudorf U, Mersch-Sundermann V, Angerer J (2007). Phthalates: toxicology and exposure. Int J Hyg Environ Health.

[18] Hsu NY, Lee CC, Wang JY, Li YC, Chang HW, Chen CY, Bornehag CG, Wu PC, Sundell J, Su HJ (2012). Predicted risk of childhood allergy, asthma and reported symptoms using measured phthalate exposure in dust and urine. Indoor Air.

[19] Just AC, Whyatt RM, Perzanowski MS, Calafat AM, Perera FP, Goldstein IF, Chen Q, Rundle AG, Miller RL (2012). Prenatal exposure to butylbenzyl phthalate and early eczema in an urban cohort. Environ Health Perspect.

[20] Kim EH, Cheong HK, Kim S, Kim YM, Lee JH, Kim KB, Jung K, Ahn KM, Lee SI. Indoor air pollution aggravates symptoms of atopic dermatitis in children. PLoS One 2015 10.1371/journal.pone.0119501 PMC4363895 25781186

[21] Kim HH, Yang JY, Kim SD, Yang SH, Lee CS, Shin DC, Lim YW (2011). Health Risks Assessment in Children for Phthalate Exposure Associated with Childcare Facilities and Indoor Playgrounds. Environ Health Toxicol.

[22] Koch HM, Preuss R, Angerer J (2006). Di(2-ethylhexyl)phthalate (DEHP): human metabolism and internal exposure-- an update and latest results. Int J Androl.

[23] Kolarik B, Naydenov K, Larsson M, Bornehag CG, Sundell J (2008). The association between phthalates in dust and allergic diseases among Bulgarian children. Environ Health Perspect.

[24] Langer S, Bekö G, Weschler CJ, Brive LM, Toftum J, Callesen M, Clausen G (2014). Phthalate metabolites in urine samples from Danish children and correlations with phthalates in dust samples from their homes and daycare centers. Int J Hyg Environ Health.

[25] Larsen ST, Hansen JS, Thygesen P, Begtrup M, Poulsen OM, Nielsen GD (2001). Adjuvant and immuno-suppressive effect of six monophthalates in a subcutaneous injection model with BALB/c mice. Toxicology.

[26] Mustieles V, Pérez-Lobato R, Olea N, Fernández MF, Bisphenol A (2015). Human exposure and neurobehavior. Nuerotoxicology.

[27] North ML, Takaro TK, Diamond ML, Ellis AK (2014). Effects of phthalates on the development and expression of allergic disease and asthma. Ann Allergy Asthma Immunol.

[28] Rudel RA, Gray JM, Engel CL, Rawsthorne TW, Dodson RE, Ackerman JM, Rizzo J, Nudelman JL, Brody JG (2011). Food packaging and bisphenol A and bis(2-ethyhexyl) phthalate exposure: findings from a dietary intervention. Environ Health Perspect.

[29] Rudel RA, Perovich LJ (2009). Endocrine disrupting chemicals in indoor and outdoor air. Atmos Environ.

[30] Schmid P, Schlatter C (1985). Excretion and metabolism of di-(2-ethylhexyl) phthalate in man. Xenobiotica.

[31] Serrano SE, Braun J, Trasande L, Dills R, Sathyanarayana S (2014). Phthalates and diet: a review of the food monitoring and epidemiology data. Environ Health.

[32] Shu H, Jonsson BA, Larsson M, Nanberg E, Bornehag CG (2014). PVC flooring at home and development of asthma among young children in Sweden, a 10-year follow-up. Indoor Air.

[33] Spanier AJ, Kahn RS, Kunselman AR, Hornung R, Xu Y, Calafat AM, Lanphear BP (2012). Prenatal exposure to bisphenol A and child wheeze from birth to three years. Environ Health Perspect.

[34] Takano H, Yanagisawa R, Inoue K, Ichinose T, Sadakane K, Yoshikawa T (2006). Di-(2-ethylhexyl) phthalate enhances atopic dermatitis-like skin lesions in mice. Environ Health Perspect.

[35] Takaro TK, Diamond M, Gobas F, Otton V, Shu H (2010). Critical review of phthalates in Canadian indoor environments.

[36] Vaidya SV, Kulkarni H (2012). Association of urinary bisphenol A concentration with allergic asthma: results from the national health and nutrition examination survey 2005–2006. J Asthma.

[37] Wang IJ, Lin CC, Lin YJ, Hsieh WS, Chen PC (2014). Early life phthalate exposure and atopic disorders in children: a prospective birth cohort study. Environ Int.

[38] Weinberg CR, Umbach DM (1999). Using pooled exposure assessment to improve efficiency in case–control studies. Biometrics.

[39] Whyatt RM, Rundle AG, Perzanowski MS, Just AC, Donohue KM, Calafat AM, Hoepner L, Perera FP, Miller RL (2014). Prenatal phthalate and early childhood bisphenol A exposures increase asthma risk in inner-city children. J Allergy Clin Immunol.

[40] Wilson NK, Chuang JC, Morgan MK, Lordo RA, Sheldon LS (2007). An observational study of the potential exposures of preschool children to pentachlorophenol, bisphenol-A, and nonylphenol at home and daycare. Environ Res.

[41] Wittassek M, Koch HM, Angerer J, Brüning T (2011). Assessing exposure to phthalates - the human biomonitoring approach. Mol Nutr Food Res.

[42] Wormuth M, Scheringer M, Vollenweider M, Hungerbuhler K (2006). What are the sources of exposure to eight frequently used phthalic acid esters in Europeans?. Risk Anal.

[43] Yamamoto T (2000). Determination of bisphenol A migrated from polyvinyl chloride hoses by GC/MS. Bunseki Kagaku.

[44] Yan H, Takamoto M, Sugane K (2008). Exposure to Bisphenol A prenatally or in adulthood promotes T(H)2 cytokine production associated with reduction of CD4CD25 regulatory T cells. Environ Health Perspect.

[45] Ye X, Pierik FH, Angerer J, Meltzer HM, Jaddoe VW, Tiemeier H, Hoppin JA, Longnecker MP (2009). Levels of metabolites of organophosphate pesticides, phthalates, and bisphenol A in pooled urine specimens from pregnant women participating in the Norwegian Mother and Child Cohort Study (MoBa). Int J Hyg Environ Health.

[46] Yu JS, Lee CJ, Lee HS, Kim J, Han Y, Ahn K, Lee SI (2012). Prevalence of atopic dermatitis in Korea: analysis by using national statistics. J Korean Med Sci.

[47] Zheng M, McErlane KM, Ong MC (2002). Hydromorphone metabolites: isolation and identification from pooled urine samples of a cancer patient. Xenobiotica.

